# Pro370Leu *MYOC* gene mutation in a large Chinese family with juvenile-onset open angle glaucoma: correlation between genotype and phenotype

**Published:** 2008-08-22

**Authors:** Ye-Hong Zhuo, Yan-Tao Wei, Yu-Jing Bai, Shan Duan, Ming-Kai Lin, H. Uri Saragovi, Jian Ge

**Affiliations:** 1 From the State Key Laboratory of Ophthalmology, Zhongshan Ophthalmic Center, Sun Yat-sen University, Guangzhou, People’s Republic of China; 2 Lady Davis Institute-Jewish General Hospital, Department of Pharmacology and Therapeutics, Oncology/Cancer Center, McGill University, Montreal, Canada

## Abstract

**Purpose:**

Glaucoma is the leading cause of irreversible blindness worldwide. Most of the cases are primary open angle glaucoma (POAG). POAG is a genetically heterogenous disease; autosomal dominance is the most frequent type of monogenic inheritance. In this study, we identified the genotype of a *MYOC* mutation and investigated the phenotype of a Chinese juvenile-onset open angle glaucoma (JOAG) pedigree (GZ.1 pedigree).

**Methods:**

Blood samples were obtained from 24 participants. We performed sequence and gene linkage analysis in the GZ.1 pedigree retrospectively. Comprehensive ophthalmologic examinations were performed for each family member. Pharmacological treatment or filtering surgery was performed as needed according to the intraocular pressure (IOP) of each individual.

**Results:**

A Pro370Leu myocilin mutation located in exon 3 of *MYOC* was identified in 24 members of the GZ.1 pedigree. Sixteen patients had juvenile-onset primary open-angle glaucoma (JOAG), and the others participating in the project had no such genotype. Analysis of polymorphic microsatellite markers indicated that the disease in GZ.1 is autosomal dominant inheritance. The patients in GZ.1 are characterized by early age of onset (before 35 years of age), severe clinical presentations, and high intraocular pressure unresponsive to pharmacological treatment; requiring 89.5% of the patients to undergo filtering surgery. Fortunately, the success rate of surgery was high. None of the patients required further medical treatment and only one demonstrated low IOP fundus changes.

**Conclusions:**

This is the first evidence of a founder effect for a Pro370Leu myocilin mutation in a Chinese POAG pedigree. The family with the Pro370Leu myocilin mutation presents with juvenile-onset glaucoma. After 10 years of follow-up, it is evident that the mutation is closely associated with the phenotype of the patients. Analysis of *MYOC* in JOAG patients may enable the identification of at-risk individuals and help prevent disease progression toward the degeneration of the optic nerve, and may also contribute to genetic counseling.

## Introduction

Glaucoma, one of the leading causes of blindness, is a chronic neurodegenerative disease that will affect over 60 million people worldwide by 2010 [1]. The disease is characterized by painless, progressive, irreversible degeneration of the optic nerve and loss of visual field. Elevated intraocular pressure (IOP) resulting from the increased aqueous outflow resistance in the trabecular meshwork is a major risk factor. Thus, pharmacological and surgical treatments aim to facilitate aqueous outflow and are essential for normalizing IOP.

Primary open-angle glaucoma (POAG) is the most common form of glaucoma, especially in North America [2], representing more than half of all cases. Although the underlying etiology of POAG is unknown, there is evidence that gene mutations can be associated with this disease. According to an epidemiological survey, about 30%–56% of patients with POAG and ocular hypertension (OHT) have a family history, and the incidence in individuals with a first degree relative having glaucoma is about 7–10 times higher than in the general population [3].

Based on age at time of diagnosis, POAG is classified as either adult- or juvenile-onset, with 35 years of age being the boundary. Most cases of POAG follow a complex pattern of inheritance, while juvenile-onset primary open-angle glaucoma (JOAG) typically shows an autosomal dominant inheritance. The phenotype of POAG is also different from JOAG. Generally, the high intraocular pressure in POAG is stable with pharmacological treatment, but JOAG is usually a much more severe disease requiring surgery to avoid loss of sight [4].

Since the first POAG-correlated mutation gene (trabecluar meshwork-inducible glucocorticoid response/myocilin; *TIGR*/*MYOC*) was identified in 1997 [5,6], there have been three genes reported to be responsible for POAG, i.e., *TIGR/MYOC*, *OPTN* (optic neuropathy inducing gene) [7,8], and *WDR36* (WD repeat domain 36 gene) [9], with *TIGR*/*MYOC* being the most frequently mutated gene [10-13]. In this case, the myocilin protein is mutated and its abnormal function increases the resistance of the aqueous humor outflow, leading to high IOP. This results in the degeneration of the optic nerve and visual field loss [14-16]. Studying the correlation between genotype and phenotype will contribute to an improved understanding of POAG.

Here, we report the analysis of *MYOC* mutations and describe clinical findings in a large Chinese autosomal dominant JOAG family (GZ.1). GZ.1 is a Pro370Leu mutation encompassing 56 family members with 19 of them exhibiting JOAG that is unresponsive to standard pharmacological treatments.

## Methods

### Subjects

The GZ.1 pedigree lives in Guangdong province, China and spans 5 generations with  56 members. The proband (III7) was tested in 1999 and diagnosed with JOAG, after which point we did an extended examination of family members. We discovered that affected individuals with documented bilateral glaucoma were present in each generation except generation V. The total number of JOAG cases is 14 (including deceased patients; I2 and II1). During follow up spanning the next 10 years, additional patients were diagnosed with JOAG; between 2000 and 2002, 2 individuals were diagnosed, and 3 more during the interval from 2003 to 2008.

 In this study, we retrospectively analyze the genotype and phenotype of the GZ.1 pedigree. The study was done in accordance with the principles of the Declaration of Helsinki. Informed parental consent, informed patient consent, and approval by the Hospital Ethics Committee (Zhongshan Ophthalmic Centre, Sun Yat-sen University) were obtained before initiating the study.

### Diagnostic criteria

The initial patient with primary open angle glaucoma (POAG) had an intraocular pressure (IOP) of 22 mmHg or higher (in absence of IOP lowering therapy), an open anterior chamber angle on gonioscopy, glaucomatous optic disc features, and visual field alteration consistent with assessed optic neuropathy. Diagnosis of juvenile-onset open angle glaucoma (JOAG) was given when patients were younger than 35 years of age at the time of POAG diagnosis. An IOP above 21 mmHg (without IOP lowing therapy) in the absence of damage to the optic nerve and loss of visual field is diagnosed as ocular hypertension (OHT).

### Clinical examination

Comprehensive ophthalmologic examinations and general medical history were taken and documented by the same two experienced doctors (Zhongshan Ophthalmic Centre, Sun Yat-sen University). The protocol included the best-corrected visual acuity with Snellen charts, slit-lamp inspection of the anterior eye, IOP measurement by Goldmann application tonometry, anterior chamber angle evaluation by gonioscopy (Goldmann), and fundus examination by 78-diopter Hruby lens including vertical and horizontal optic cup disc ratio (C/D ratio) assessment. All subjects underwent automated visual field examination (tested with Humphrey, SITA fast strategy, program 30–2). The Optical Coherence Tomography (OCT) and color fundus photographs of the disc and macula were tested to aid with assessment of the patient’s visual condition and stage of illness.

### Genomic DNA extraction

Peripheral blood leukocytes were obtained from all available family members, including 16 affected and 8 unaffected individuals. Genomic DNA was extracted from peripheral blood as recommended in the QIAamp DNA Blood Max Kit (QIAGEN, Hilden, Germany).

### Microsatellite marker analysis

A genome-wide scanning was performed using a set of fluorescence-labeled microsatellite markers spanning the entire human genome at approximately 10 cM intervals with the ABI PRISM Linkage Mapping Set MD-10 (Applied Biosystems Inc.). DNA samples were subjected to polymerase chain reaction (PCR) amplification with a standard cycling profile of 30 cycles at 94 °C for 30 s, 55 °C for 30 s, and 72 °C for 45 s. PCR reactions were performed in a 10 μl volume containing 0.4 mM of each primer, 200 μM dNTPs, and 1U Taq DNA polymerase. The PCR products were separated on 5% denaturing polyacrylamide gel in an Applied Biosystems 377 DNA sequencer (Applied Biosystems Inc.).

### Linkage analysis

Linkage analysis was performed by calculating two-point lod scores using the MLINK routine from LINKAGE (ver. 5.1) software suite (provided in the public domain by the Human Genome Mapping Project Resources Center, Cambridge, UK). LOD scores were calculated with a presumed penetration rate of 95% and an allele frequency of 0.001 for the disease allele.

### Mutation screening

To examine the probability of the existence of disease-associated mutations, exon-specific primers were designed for the myocilin gene (GenBank AB006686). PCR amplification was performed in a GeneAmp PCR System 9700 (Applied Biosystems Inc.), with 100 ng of genomic DNA, 10 pmol of primers and 1U of Taq DNA polymerase in a reaction volume of 20 μl. Primer sequences and their PCR product sizes are given in Table 1. Samples were subjected to a PCR amplification protocol beginning with a denaturation step at 94 °C for 2 min, followed by 30 cycles, each consisting of a denaturation step at 94 °C for 30 s, an annealing step at about 55 °C-58 °C for 30 s, and an extension step at 72 °C for 30 s, followed by a final extension at 72 °C for 8 min. The amplified exons were purified and sequenced on an automated DNA sequencer (model 377; Applied Biosystems Inc.). All PCR products were sequenced in both forward and reverse directions.

**Table 1:** Sequences of primers used for mutation screening of myocilin gene.

**Name**	**Primer sequence**	**Product** **Length** **(bp)**
E1A	5′-TATTTTCTAAGAATCTTGCTGG-3′	394
	5′-TGGATTCATTGGGACTGG-3′	
E1B	5′-GAAGCCTCACCAAGCCTC-3′	342
	5′-GCCTGGTCCAAGGTCAAT-3′	
E1C	5′-CTGGAGGCCACCAAAGCT-3′	448
	5′-AGAAAGGGCAGGCAGGGA-3′	
E2	5′ CATAGTCAATCCTTGGGC-3′	392
	5′-CTGCAGACCTGCTCTGACAA-3′	
E3A	5′-TTTCTGAATTTACCAGGATG-3′	426
	5′-GTCAATGTCCGTGTAGCC-3′	
E3B	5′-CGGACAGTTCCCGTATTC-3′	431
	5′-GCTTGGAGGCTTTTCACA-3′	
E3C	5′-CAAGACCCTGACCATCCC-3′	412
	5′-TGCCCCAAATCACAAGAA-3′	

### Clinical management

Topical medication was given to patients with an IOP higher than 21 mmHg. Patients whose IOP could not be controlled with medicine underwent combined trabeculectomy. After surgery, patients were followed closely until their IOP was stable. The assessment included: the best-correct visual acuity, IOP, the depth of the anterior chamber, filtering bleb morphous, C/D ratio, visual field, and OCT.

## Results

### Genotype of the GZ.1 pedigree

**Figure 1:**
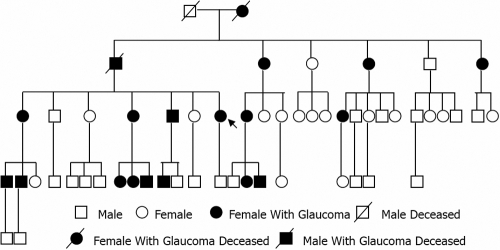
Pedigree structure of the Chinese family with 19 affected subjects in 5 generations. Squares indicate male subjects; circles, female subjects; solid symbols, affected family members with JOAG; unfilled symbols, unaffected family members; diagonal line, deceased individual. Arrow indicates the proband (III:7). According to the distribution of the affected members, the heredity of the GZ.1pedigree is autosomal dominant.

A *MYOC* Pro370Leu mutation was identified in 16 affected individuals of the GZ.1 pedigree, and the rate of occurrence of mutation was 100%. According to the distribution of the affected family members (Figure 1), the heredity of the GZ.1 pedigree is autosomal dominant.

**Figure 2:**
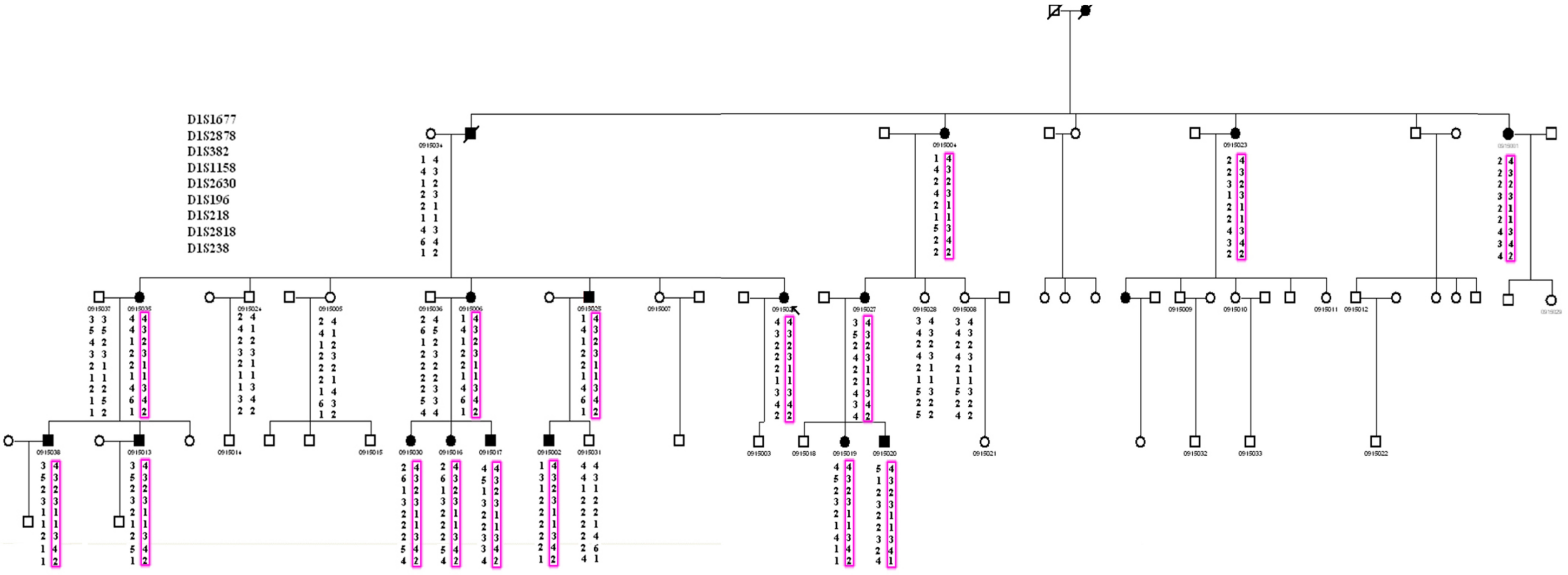
Haplotype analysis of the GZ.1 pedigree using microsatellite markers encompassing *MYOC* on the long arm of chromosome 1. *MYOC* resides between markers D1S196 and D1S2818. Square cells refer to males and circle cells refer to females; Filled cells are JOAG; diagonal line, deceased; and arrow, proband. The analysis was performed in 24 informative family members including 16 affected and 8 unaffected individuals. Segregating haplotypes are shown in the rectangles.

**Figure 3:**
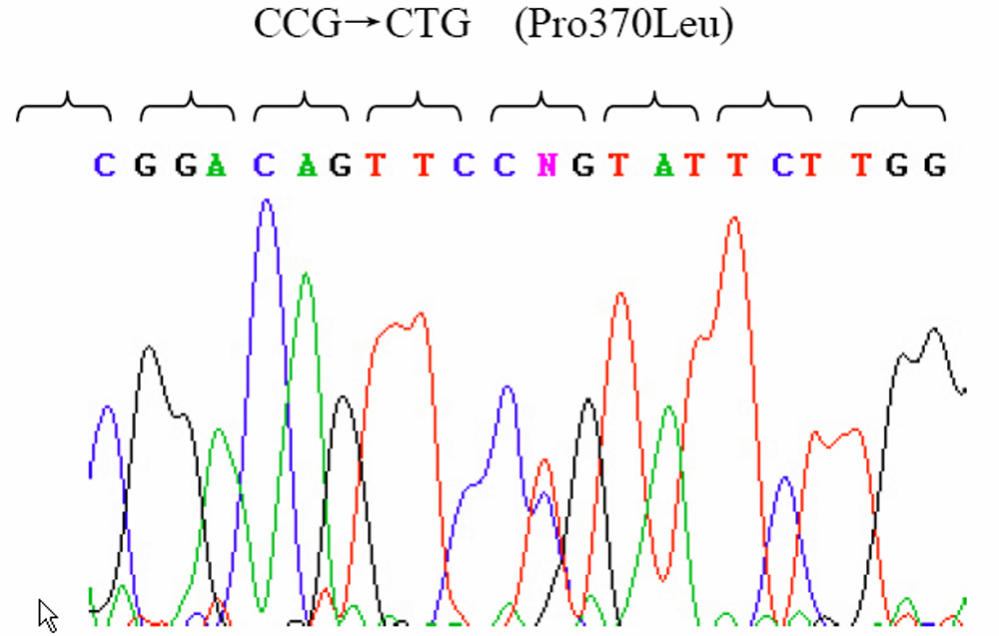
Detection of the Pro370Leu *MYOC* mutation by direct polymerase chain reaction DNA sequencing in the GZ.1 pedigree. Representative chromatogram contains sequence from the noncoding DNA strand. The location of the mutation within *MYOC* and the nature of the nucleotide change (C->T ) were shown as a double peak in the heterozygous condition.

Linkage analysis and Haplotype analysis demonstrated that all affected individuals were heterozygous for this change (Figure 2). Two-point LOD scores of markers in this region are summarized in Table 2. A maximum LOD score of 5.46 was found for marker D1S2818 at 0.0. This marker is located in the close vicinity of *MYOC*. Mutation analysis of this gene showed a heterozygous C->T transversion at nucleotide 1,109 in exon 3, resulting in a substitution of Proline to Leucine (Pro370Leu; Figure 3). This mutation cosegregated in all affected individuals and was not observed in unaffected subjects.

**Table 2:** Two-Point LOD Scores of the 9 DNA Markers in the genome-wide scan of the GZ.1 pedigree.

**Marker**	**Two-point LOD score values at recombination fraction (θ=)**						**Zmax**	**θmax**
	**0.0**	**0.1**	**0.2**	**0.3**	**0.4**	**0.5**		
D1S1677	-4.21	1.37	1.38	1.00	0.45	0	1.38	0.2
D1S 2878	4.82	3.98	3.05	2.03	0.93	0	4.82	0.0
D1S 382	1.52	1.25	0.96	0.65	0.32	0	1.52	0.0
D1S 1158	-1.51	1.39	1.31	0.98	0.54	0	1.39	0.1
D1S 2630	1.85	1.62	1.09	0.51	0.09	0	1.85	0.0
D1S 196	-2.37	0.50	0.52	0.34	0.11	0	0.52	0.2
D1S 218	3.00	2.88	2.38	1.65	0.78	0	3.00	0.0
D1S 2818	5.46*	4.52	3.48	2.34	1.09	0	5.46	0.0
D1S 238	-5.5	0.84	0.80	0.59	0.32	0	0.84	0.1

Microsatellite markers with an average spacing of 10 cM were used in the initial genome-wide scan. The asterisk indicates the promising chromosome region on 1q21-q23 within a marker presented a suggestive LOD score value of Zmax=5.46 at θ=0.00.

Using a computer sequence alignment program (BLAST), amino acid sequences of *MYOC* obtained from GenBank were compared among human, rat, mouse, bovine and fugu. The comparison revealed that Pro370Leu occurred at a highly conserved position of the myocilin gene (Table 3).

**Table 3:** Comparison of amino acid sequences of myocilin between human, rat, mouse, bovin, fugu, and dare. The result revealed Pro370Leu occurred at highly conserved positions (bold **P**).

**Protein**	**Amino acid 370**
Myoc_human	ETVKAEKEIPGAGYHGQF **P** YSWGGYTD
Myoc_rat	ETVKAEKEIPGAGYHGQF **P** YAWGGYTD
Myoc_mouse	ETVKAEKEIPGAGYHGHF **P** YAWGGYTD
Myoc_bovin	ETLKAEKEIPGAGYHGQF **P** YSWGGYTD
Myoc_fugu	ESLAARLDLPHAGFHGQH **P** YSWGGYTD
Myoc_dare	ESIAARRDLPHAGFHGQF **P** YSWGGYTD

### Clinical phenotype of the GZ.1 pedigree

We studied an autosomal dominant family (GZ.1 pedigree) with 17 POAG patients; 31.48% of all family members. Among the affected individuals, 6 of are male and 11 are female. I2 and II1 were deceased at the time of our study, but their medical records provided adequate information concerning their ocular disease.

**Figure 4:**
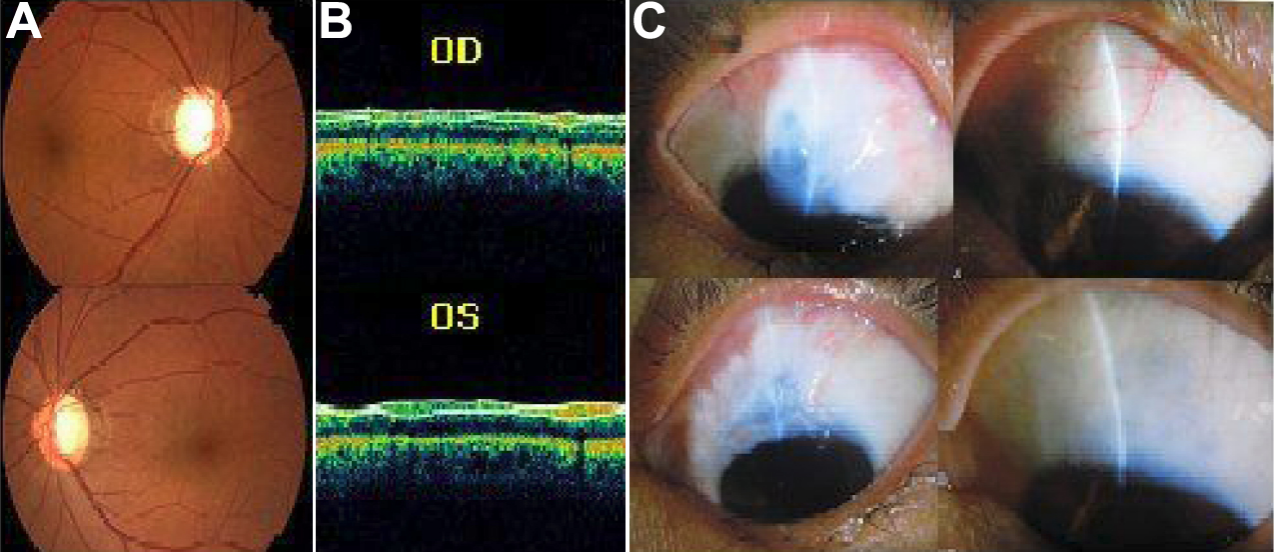
Individual III5 (top) right eye, (bottom) left eye. **A**: Fundus images showing typical glaucomatous cupping of the optic disc, **B**: OCT images showing thinner nerve fiber layer, **C**: Thin-walled filtering blebs after filtering surgery.

The onset of disease with all these patients was insidious. The average age at diagnosis was 30 years (ranging from 11 to 35 years), and the mean IOP before medical or surgical care at the time of last follow up was 45.52±6.39 mmHg (range from 35 to 56 mmHg). All of the patients exhibited severe degeneration of the optic nerve and visual field defects (Figure 4). According to our investigation, most of the JOAG patients were unresponsive to antiglaucoma medications; filtering surgery was often required for long-term IOP control. At the time of the study, 17 patients (34 eyes) were operated on to control IOP with combined trabeculectomy; none of them needed a second operation. All of the surgeries were done in the Zhongshan Ophthalmic Center, Sun Yat-sen University by experienced doctors. One OHT patients (IV21) was under treatment with medication.

After surgery, all participants obtained their target IOP; none of them requires any medications and all appear to be prone to filtering bleb scarring. It is gratifying that the postoperative IOP in both eyes of each patient has been controlled at around 10 mmHg without any severe complications, with the exception of one patient with low IOP fundus changes.

During the 10 years of follow up with the GZ.1 pedigree, we discovered an unusual phenomenon: with the passage of each generation, the age of onset tended to be younger, the pathogenetic condition tended to be more severe, and the post-operation IOP declined further. All of generation II and III patients exhibited type II filtering blebs, while all of generation IV patients exhibited type I filtering blebs (Figure 4). The condition of the patients at the last follow up can be seen in Table 4.

**Table 4:** Phenotype characteristic of the 17 patients with JOAG from GZ.1 pedigree at last follow up.

**Subject number**	**Age**	**Gender**	**BCVA**		**IOP (mmHg)**		**C/D**				**VF**	
			**RE**	**LE**	**RE**	**LE**	**RE**		**LE**		**RE**	**LE**
							**V**	**H**	**V**	**H**		
II2	74	F	0.3	NLP	17.1	17.5	0.8	0.8	0.9	0.9	N/A	N/A
II4	65	F	0.3	0.5	7	22.6	0.6	0.7	0.9	0.8	PC	IZD
II6	58	F	1.0	1.2	15.6	12.1	0.4	0.5	0.6	0.7	N/A	N/A
III1	59	F	0.4	0.7	16.3	13.7	0.9	0.8	0.9	0.8	N/A	N/A
III4	48	F	1.5	1.2	27.3	23	0.5	0.5	0.7	0.7	N	N
III5	44	M	LP	1.2	19	18	1.0	1.0	0.8	0.7	N/A	N/A
III7	38	F	1.5	0.1	14	15.4	0.4	0.4	1.0	0.9	N/A	N/A
III8	55	F	1.0	0.6	13	16	0.5	0.6	0.95	0.85	N/A	N/A
III19	35	M	1.5	1.2	15.2	17.9	0.2	0.2	0.2	0.2	N/A	N/A
IV1	37	M	0.1	LP	16	19	0.9	1.0	1.0	1.0	N/A	N/A
IV2	36	M	1.5	1.5	14	19	0.2	0.2	0.4	0.5	N/A	N/A
IV8	26	F	1.0	1.2	11	19	1.0	1.0	0.9	0.9	IZD	IZD
IV9	21	F	1.5	1.5	28	26	0.3	0.4	0.5	0.5	N	N
IV10	19	M	1.5	1.2	36	37	0.9	0.8	0.55	0.6	IZD	N
IV11	18	M	1.5	HM	8	7	0.5	0.4	0.3	0.3	N/A	N/A
IV16	31	F	1.0	0.9	10	16	0.5	0.5	1.0	1.0	N/A	N/A
IV17	32	M	0.5	0.6	8	8	0.8	0.8	0.8	0.8	N/A	N/A

The Pro370Leu mutation was present in all subjects in the table. All patients in the table had undergone trabeculectomy surgery to both eyes. Abbreviations in the table are: JOAG; juvenile open-angle glaucoma, BCVA; best-correct visual activity, RE; right eye, LE; left eye, IOP; intraocular pressure, C/D; cup disk ratio, V; vertical, H; horizontal, VF; visual field, F; female, M; male, OU; both eyes, N; normal, IZD; indicated inferior zone defect, NLP; no light perception, LP; light perception, and HM; hand movement, PC; poor cooperation, N/A; unavailable.

## Discussion

*MYOC* was the first gene in which mutations were found to cause glaucoma. *MYOC* mutations account for most dominant juvenile glaucoma cases and for approximately 2% to 4% of unselected adult onset POAG [6]. More than 70 missense mutations in *MYOC* have been identified, with the majority of them being clustered in the conserved olfactomedin domain of exon 3 [17,18]. In this study, we report linkage of autosomal dominant open-angle glaucoma in a large Chinese family to a region on chromosome 1 between D1S218 and D1S2818. A missense mutation (Pro370Leu) in exon 3 of *MYOC*, one of the candidate genes mapped in this region, was identified in this family to cosegregate with the glaucoma phenotype. No other sequence changes were found in the entire coding region or splice junctions of *MYOC* in this family. All subjects with a diagnosis of POAG had this mutation. It is reasonable to decide, then, that this variant is pathogenic. The findings in the current study enrich the evidence for *MYOC* as a causative gene for POAG.

It has been observed that there might be some correlation between clinical phenotypes and different mutations in *MYOC*. A nonsense mutation Gln368STOP, the most frequently identified GLC1A mutation, is associated with late-onset POAG. The later age at diagnosis of 52 years and the lower mean peak IOP of 28 mmHg suggest that the Gln368STOP mutation gives rise to a more mild phenotype than mutations associated with juvenile open-angle glaucoma [19]. The Thr377Met mutation results in a more severe phenotype of the disease than the Gln368Stop mutation. Wiggs et al. [20] described a family in which the proband was diagnosed at age 42 years with an IOP at diagnosis of 24 mmHg. Shimizu et al. [21] described a family with a mean age at diagnosis of 38 years and a mean maximum IOP of 44 mmHg.

It is noteworthy that the Pro370Leu mutation has been reported to be widely distributed and found by multiple research groups in different locations, with data demonstrating juvenile-onset glaucoma in French, Japanese, North American, German, and Indian families [22-25]. JOAG is an uncommon autosomal dominant form of glaucoma. Onset occurs most often before the fourth decade of life and the phenotype is clinically more severe than late-onset POAG. Most of the reported pedigrees linked to Pro370Leu had the following characteristics: (1) development of POAG at a very early age, (2) high peak levels of IOP, and (3) poor response to medical treatment [22-25]. The phenotype of POAG associated with the Pro370Leu mutation was thoroughly assessed in our data set. The mutation carriers with glaucoma seen in this study had a form of early adult-onset glaucoma associated with elevated IOP. The age at diagnosis of POAG in mutation carriers ranged from 11 to 35 years (mean 30 years). Without medication, the patients with mutation presented greater median IOP of 45.52±6.39 mmHg. Most of the patients were unresponsive to antiglaucoma medications and filtering surgery was usually required for long-term IOP control. Thus the Pro370Leu mutation in this Chinese family presents a similar clinical phenotype as previously reported. However, there are some new clinical features; the age of onset became younger with the passage of each generation. Carriers were 44.5 years (range, 36–56 years) in generation II, while in generation III the mean age at diagnosis in 11 cases was 34.4 years (range, 30–44 years). Therefore, we believe that Pro370Leu might represent a severe and strong disease allele in Chinese peoples, exhibiting an earlier onset and more aggressive glaucoma phenotype.

So far, little has been known about the exact roles played by myocilin in the development of POAG. Current studies show that the mutant myocilin is not correctly folded into a functional conformation and accumulates into aggregates inside TM cells [26-28]. According to the structure of myocilin, Pro370Leu is located within the highly conserved OLF-domain of this protein, a major component of the extracellular matrix of the olfactory neuroepithelium [28]. Interestingly, this region contains most the reported mutations identified in patients with POAG. These factors together suggest that the domain is very important for the function of this protein.

Based on our evidence, we believe that genotyping will have predictive value, at least in cases analogous to the GZ.1 pedigree, where all of the affected patients have the Pro370Leu mutation, which can then serve to predict JOAG. The early detection of the at-risk individual, will allow the adoption of optimal measures to prevent the progress of the disease [29].

In conclusion, our data provide strong evidence of a founder effect for the Pro370Leu *MYOC* mutation in a Chinese family and show that the genetic analysis of this mutation could play a key role in the management of autosomal dominant JOAG in affected families from this country. The genetic analysis of *MYOC* in this family could not only be used to identify at-risk persons decades before the disease manifests phenotypically, but also to aid in genetic counseling. Next, we will use the genetic mutations in an animal model to explore further the mechanisms of disease.
